# Stigma toward Schizophrenia among Parents of High School Students

**DOI:** 10.5539/gjhs.v5n6p46

**Published:** 2013-08-14

**Authors:** Hatsumi Yoshii, Yuichiro Watanabe, Atiqul Haq Mazumder, Hideaki Kitamura, Kouhei Akazawa

**Affiliations:** 1 School of Health Sciences, Faculty of Medicine, Tohoku University, Miyagi, Japan; 2 Department of Psychiatry, Niigata University Graduate School of Medical and Dental Sciences, Niigata, Japan; 3 Division of Medical Education, Comprehensive Medical Education Center, School of Medicine, Faculty of Medicine, Niigata University, Niigata, Japan; 4 National Institute of Mental Health, Dhaka, Bangladesh; 5 Department of Medical Informatics and Statistics, Niigata University Graduate School of Medicine, Niigata, Japan

**Keywords:** schizophrenia, stigma, high school students, parents

## Abstract

Stigma toward schizophrenia is an important area of research as it is frequently a barrier to early intervention. This study attempted to identify factors underlying stigma in Japan. Because even adolescents can develop schizophrenia, 357 Japanese parents of high school students were enrolled. All parents lived outside the areas affected by the Tohoku earthquake that occurred on March 11, 2011 (ie, parts of Iwate, Miyagi, and Fukushima prefectures). Factor analysis using the Link Devaluation–Discrimination Measure identified two factors: comparison with an able-bodied person and non-comparison with an able-bodied person. Regression analysis revealed that family structure had independent effects on factor 2 (p <0.05), and ANOVA showed that education had independent effects on factor 2 (p <0.05). These results suggest that education programs that seek to counteract stigma should target curricula in high schools and vocational schools.

## 1. Introduction

Schizophrenia can develop in high school student (Nishida et al., 2008). However, stigma reduces the chance for early intervention in schizophrenia (Addington et al., 2012; Tanaka et al., 2003; Lysaker et al., 2007; Esterberg et al., 2008). The possibilities of earlier detection and early treatment of schizophrenia might be increased if stigma can be reduced among parents of high school students (Perlick et al., 2001). Stigma toward schizophrenia has been studied in many populations (Loch et al., 2013; Broussard et al., 2012; Scior et al., 2013), including Brazil (Loch et al., 2013), an African-American community (Broussard et al., 2012), and across ethnic groups in the United Kingdom (white, Asian, and black African UK residents) (Scior et al., 2013). However, these studies did not investigate early intervention.

Public stigma and discrimination have harmful effects on the lives of people with serious mental illnesses (Corrigan et al., 2012), as they can lead to discrimination in education, employment, personal relationships, marriage, and housing (Takahashi et al., 2009). The future of an adolescent with a diagnosis of a serious mental disorder would thus appear to be unpromising. Researchers in psychiatry must seek to identify interventions that effectively address such stigma. In addition, sociocultural milieu can influence stigma toward schizophrenia (Thirthalli et al., 2012). Increased resistance to stigma requires an understanding of one’s own sociocultural background and a strong social network, which provide a sense of comfort and security that enable a fulfilling life (Tang et al., 2012). Therefore, researchers in Japan must consider the characteristics of stigma toward schizophrenia that are unique to this country. In addition, early intervention must be considered.

Previous Japanese studies found evidence of self-stigma among people with schizophrenia (Uchino et al., 2012), stigma among psychiatric staff (Hanzawa et al., 2012; Hori et al., 2011), and a change in attitudes toward the disease after the official Japanese term used for schizophrenia was changed (Umehara et al., 2011). Previous studies also identified factors associated with early intervention for schizophrenia, including the attitudes of parents of junior and senior high school students (Yoshii et al., 2011), the impact of changing the term used for schizophrenia in Japan (due to stereotypical beliefs regarding schizophrenia) (Takahashi et al., 2009), and attitudes toward schizophrenia among Japanese and Taiwanese elementary school teachers (Kurumatani et al., 2004). The relation between stigma and early intervention in schizophrenia has unfortunately not been adequately studied in Japan. Such research is necessary, however, if effective early interventions for schizophrenia are to be developed. We attempted to identify the factors underlying stigma among parents of high school students in Japan.

## 2. Methods

### 2.1 Participants

For the above-mentioned reasons, 357 parents (enrolled by a Japanese company specializing in research recruitment) of high school students were asked to complete a questionnaire. Parents living in the area affected by the Japanese Tohoku earthquake of March 11, 2011 were excluded (parts of Miyagi, Fukushima, and Iwate prefectures). This study was approved by the Ethics Committee of the Niigata University School of Medicine.

### 2.2 Measurement

The questionnaire collected information on sociodemographic data and general attitudes toward schizophrenia, which was evaluated based on our modification of the Link Devaluation–Discrimination Measure (Link 1987). In brief, questionnaire references to a ’patient in a psychiatry department’ were changed to a ’patient with schizophrenia’. This scale consists of 12 items that were graded using a 4-point Likert scale (1-4 points), with higher scores representing increased stigma. Items 1, 2, 3, 4, 8, and 10 were reverse-scored.

### 2.3 Statistical Analysis

All analyses were performed by using the Statistical Package for Social Sciences (SPSS) version 18.0. All statistical tests were two-tailed, and a p-value less than 0.05 was considered to indicate statistical significance. Factor analysis with the major factor method and Promax rotation was used to examine the factor structure for stigma. The reliability of scales with multiple items was evaluated by Cronbach’s α. The t test was used to analyze differences in the distributions of factor scores between categories when the factor had two categories, and ANOVA was used when the factor had more than two categories. The relative importance of different sociodemographic variables in predicting factor scores was examined by multiple regression analysis.

## 3. Results

### 3.1 Participant Characteristics

The participants were 357 Japanese parents (age range, 37-62 years) of high school students; 192 (53.8%) were male and 165 (46.2%) were female. Regarding education status, 153 (42.9%) had a university education and 105 (29.4%) had a high school education. The most frequently reported birthplace was the Kanto region (n=113; 31.7%).

### 3.2 Link Devaluation–Discrimination Measure Scores in Parents of High School Students

Mean (SD) score on the Link Devaluation–Discrimination Measure was 32.55 (4.42); the score range was 12 to 48. The Cronbach’s α of 0.788 for the Link Devaluation–Discrimination Measure was considered acceptable. The median score was 32.00, which indicates moderate stigma. Results of factor analysis of the Link Devaluation–Discrimination Measure revealed two factors with eigenvalues ≥1. Factor 1 was comparison with an able-bodied person (proportion of variance, 32.0%; α coefficient, 0.82), and factor 2 was non-comparison with an able-bodied person (proportion of variance, 17.3%; α coefficient, 0.73), for a total variance of 19.5%. The mean score was 16.98 for factor 1 (SD, 2.79; range, 6-24) and 15.57 for factor 2 (SD, 2.76; range, 6-24; Table 1).

**Table 1:** Factor loading for the link devaluation–discrimination measure (revised) with major factor method and promax rotation

Factors and items	Factor 1	Factor 2
**Factor 1: Comparison with able-bodied person**		
Q1: Most people would accept a person with schizophrenia as a close friend.	0.676	0.041
Q2: Most people believe that a person with schizophrenia is as intelligent as the average person.	0.75	-0.008
Q3: Most people believe that a person with schizophrenia is just as trustworthy as the average citizen.	0.863	-0.064
Q4: Most people would accept a person who had fully recovered from schizophrenia as a teacher of young children in a public school.	0.591	-0.052
Q8: Most employers will hire a person with schizophrenia if he or she is qualified for the job.	0.572	0.027
Q10: Most people in my community would treat a person with schizophrenia just as they would treat anyone.	0.519	0.05
**Factor 2: Non-comparison with able-bodied person**		
Q5: Most people feel that becoming schizophrenic is a sign of personal failure.	0.078	0.364
Q6: Most people would not hire a person with schizophrenia to take care of their children, even if he or she had been well for some time.	0.03	0.602
Q7: Most people think less of a person who has had schizophrenia.	-0.011	0.657
Q9: Most employers will pass over the application of a person with schizophrenia in favor of another applicant.	-0.215	0.443
Q11: Most young women would be reluctant to date a man who has schizophrenia.	0.02	0.654
Q12: Once they know a person had schizophrenia, most people will take his or her opinions less seriously.	0.094	0.675
**Eigenvalue**	3.837	2.078
**Explained variance %**	31.971	17.317
**α**	0.822	0.732

Factor loadings >0.3 are used. Total variance, 19.501%; α = 0.788. Q1, 2, 3, 4, 8, and 10 were reversed items and were reverse-scored.

### 3.3 Link Devaluation–Discrimination Measure Scores by Factor and Demographic Characteristics

The mean scores for factors 1 and 2, by demographic characteristic, are shown in Table 2. Education was significantly associated with the score for factor 2 (p=0.02). A high school education was associated with the highest score for factor 2 (mean, 16.06), and a junior high school education was associated the lowest score for that factor (mean, 11.0). The association between birthplace and factor 1 was of borderline statistical significance (p=0.051). Other demographic factors (eg, gender, age, and domicile) were not significantly associated with scores for factors 1 and 2 (p >0.05). Multiple regression analysis revealed that family structure was significantly associated with factor 2 (p <0.05; Table 3).

**Table 2:** Factor scores

	n	factor 1		factor 2		

		mean	p	mean	p
**Gender**			p=0.856		p=0.521
Male	192	17.01		15.48	
Female	165	16.95		15.67	
**Age, years**			p=0.454		p=0.064
30 - 39	14	16.86		15.57	
40 - 49	239	17.14		15.49	
50 - 59	103	16.62		15.8	
60 - 69	1	18		12	
**Education level**			p=0.356		p=0.02
Junior high school	2	15		11	
High school	105	17.2		16.06	
Vocational school	42	16.67		16.05	
Junior college	40	17.43		14.8	
University	153	16.76		15.43	
Graduate school	14	17.86		15.07	
Other	1	14		13	
**Birthplace**			p=0.051		p=0.702
Hokkaido	6	15.83		15	
Tohoku	4	17.75		17.25	
Kanto	113	17.17		15.72	
Sinetsu	10	15		16.2	
Hokuriku	10	15.1		14.4	
Tokai	53	17.49		15.25	
Kinki	90	16.68		15.46	
Chugoku	21	17.33		15.86	
Shikoku	18	18.17		16.33	
Kyusyu	31	16.74		15.42	
Okinawa	1	17		14	
**Domicile**			p=0.347		p=0.982
Kanto	135	17.01		15.56	
Sinetsu	8	14.75		15.5	
Hokuriku	6	15.67		14.17	
Tokai	52	17.23		15.69	
Kinki	93	16.89		15.59	
Chugoku	21	17.19		15.48	
Shikoku	12	18		16	
Kyusyu	28	17.04		15.57	
Okinawa	2	16		15.5	
**Marriage status**			p=0.295		p=0.070
Unmarried	3	17.67		18.33	
Married	338	16.93		15.5	
Divorced	16	18		16.56	
**Family structure**			p=0.583		p=0.076
2 parents	280	16.9		15.41	
1 parent	14	17.36		16.57	
3 generations	56	17.06		15.91	
Others	7	18.14		17.43	
**Employment status**			p=0.185		p=0.959
Full-time	184	17.01		15.53	
Part-time	67	17.13		15.67	
Self-employed/housework/liberal profession	39	16.59		15.67	
Side job					
Full-time homemaker	62	16.89		15.47	
Unemployed	4	19.5		15.75	
Other	1	12		18	
**Family income, yen**			p=0.813		p=0.272
<1 million	9	16.56		14.78	
1 to 3 million	32	17.25		16.41	
3 to 5 million	70	16.69		15.3	
5 to 10 million	178	17.09		15.47	
>10 million	68	16.93		15.82	
**Proximity to person with schizophrenia**			p=0.428		p=0.186
Yes	24	17.42		16.29	
No	333	16.95		15.52	
**Participation in welfare activities for people with mental illnesses**			p=0.813		p=0.114
Yes	30	17.03		16.33	
No	327	16.97		15.5	
t-test, ANOVA					

**Table 3:** Results of multiple regression analysis of factors 1 and 2 as dependent variables (n = 357)

**Variable**	**Factor 1**			**Factor 2**		

	β	t	p	β	t	p
**Gender**	-0.01	-0.181	0.856	0.034	0.643	0.521
**Age**	-0.064	-1.205	0.229	0.031	0.586	0.558
**Education**	-0.028	-0.527	0.599	-0.101	-1.918	0.056
**Birthplace**	0.016	0.304	0.761	-0.018	-0.346	0.73
**Domicile**	0.027	0.509	0.611	0.012	0.232	0.817
**Marriage status**	0.072	1.364	0.173	0.056	1.057	0.291
**Family structure**	0.061	1.154	0.249	0.113	2.139	0.033
**Employment status**	-0.015	-0.278	0.781	0.004	0.081	0.935
**Job**	-0.011	-0.192	0.848	0.008	0.142	0.887
**Family income**	0.012	0.223	0.824	0.004	0.084	0.934
**Proximity to person with schizophrenia**	-0.042	-0.793	0.428	-0.07	-1.326	0.186
**Participation in welfare activities for people with mental illness**	-0.017	-0.313	0.754	-0.084	-1.584	0.114

β: standardized regression coefficient; t: t-value; R: multiple correlation coefficients

## 4. Discussion

Early diagnosis (during the prodromal phase) and early treatment of the first episode of psychosis (FEP) may prevent or reduce disease morbidity (Lenciu et al., 2010). Treatment is often delayed, even in developed nations such as the United Kingdom, and it may require as long as 2 years from the first signs of psychosis for families to seek help (Kulhara et al., 2008). Mental disorders are common in young people; yet many do not seek help (Anzai et al., 2002). Studies of help-seeking have shown that stigma components have varying effects on help-seeking (Wright et al., 2011). We hypothesize that decreasing stigma to schizophrenia among parents of high school student would lead to earlier detection and early treatment of schizophrenia. We therefore limited our investigation of stigma to parents of high school students, and the factor of stigma was investigated. Stigma is an overarching term that comprises three key elements: problems of knowledge, problems of attitudes, and problems of behavior (Durand-Zaleski et al., 2012). The present results revealed two important factors: comparison with an able-bodied person and non-comparison with an able-bodied person. The former is synonymous with public stigma, which was first conceptualized by Corrigan and Watson (Peluso et al., 2010). In a probabilistic sample of 500 individuals aged 18 to 65 years and living in the city of Sao Paulo, Brazil, Peluso and Blay found that 59.0% perceived people with schizophrenia as capable of arousing negative reactions and 57.2% as capable of arousing discrimination in society (Peluso et al., 2011). Stigma components associated with reduced willingness to seek help for a mental disorder include personally believing that a person is weak not sick, social distance, perceived stigma, and self-stigma (Wright et al., 2011).

High school students hold certain stereotypical beliefs about people with schizophrenia and are sometimes reluctant to interact with them (Economou et al., 2012). However, upon completion of an intervention, positive changes were seen in student beliefs, attitudes, and desired social distance, and these changes in beliefs and attitudes were still present one year later (Economou et al., 2012). Imaging of intergroup contact can combat mental health stigma by reducing anxiety, avoidance, and negative stereotyping (Stathi et al., 2012). The present authors believe that interventions should also target parents of students, as parents of children with schizophrenia typically have difficulties in understanding their child’s condition. Using unit-factor analysis, we previously sought to identify factors influencing stigma against schizophrenia among parents of junior high and high school students (Yoshii, 2011). The extracted factors were occupation, household annual income, history of contact with a person with schizophrenia, and history of participating in a mental-health welfare activities. These results obviously differ from those of the present study. The above-mentioned factors were not significantly associated with scores for factors 1 or 2 (p >0.05) in the present study. Therefore, it can be said that stigma differs according to the subject.

The stigma associated with mental illness in Japanese families is high (Anzai et al., 2002), and individuals with schizophrenia and their families have suffered greatly from the effects of such stigma (Omori et al., 2012). Individuals with mental illness who are highly stigmatized face serious obstacles in multiple domains, including social isolation, loss of income, difficulty obtaining housing and employment, depression, decreased quality of life, and reduced access to medical care (Park et al., 2013). The quality of life of patients with schizophrenia is lower than that of the general population and that of individuals with certain physical disorders or other mental illnesses (Sibitz et al., 2011). Thus, the study of factors associated with public stigma is important in understanding the construction of stigma and in developing anti-stigma strategies aimed at the population (Peluso et al., 2010). Patients with schizophrenia were compared with an able-bodied person in our analysis of parents of high school students. Family structure and education had independent effects on the factor of non-comparison with an able-bodied person. In a study of relatives of people with schizophrenia, opinions and attitudes regarding schizophrenia were related to education level, economic status, and geographic origin (Bouhlel et al., 2012). These results suggest that education programs that seek to counteract stigma should target high school and vocational school curricula.

Strategies have been developed to reduce stigma among people with mental illnesses. Targeted strategies to decrease stigma toward people with schizophrenia may be useful to facilitate their social participation and full inclusion in the community. However, such strategies must conform to the characteristics of the target community. Therefore, it is necessary to understand the demographic characteristic of the target community (eg, educational level). The present authors hope to develop relevant strategies in Japan. In Japan, educational level is lower among rural communities than in cities (Benesse Educational Research and Development Institute). Therefore, anti-stigma measures that target parents of high school students in Japan, especially households with one parent and those in rural communities, might be important. Finally, community-level programs and the participation of psychiatric specialists could help individuals and their families overcome the challenges of mental illness and improve their quality of life (Lenciu et al., 2010).
